# Prevention of disease progression in *Leishmania infantum*-infected dogs with dietary nucleotides and active hexose correlated compound

**DOI:** 10.1186/s13071-018-2705-z

**Published:** 2018-02-21

**Authors:** Sergi Segarra, Guadalupe Miró, Ana Montoya, Luis Pardo-Marín, Joan Teichenné, Lluís Ferrer, José Joaquín Cerón

**Affiliations:** 1 R&D Bioiberica S.A.U, pl. Francesc Macià 7, 08029 Barcelona, Spain; 2 Department of Animal Health, Veterinary Faculty, Universidad Complutense de Madrid, Avda. Puerta de Hierro s/n, 28040 Madrid, Spain; 3 Interlab-UMU, Campus de Excelencia “Mare Nostrum”, University of Murcia, Campus Espinardo, 30071 Murcia, Spain; 4 Eurecat - Health and Nutrition Unit, TECNIO, CEICS, av. Universitat 1, 43204 Reus, Spain; 5 Department of Clinical Sciences, Tufts Cummings School of Veterinary Medicine, 200 Westboro Road, North Grafton, MA 01536 USA

**Keywords:** *Leishmania infantum* infection, Disease progression, Canine leishmaniosis, Dietary nucleotides, AHCC, Clinically healthy infected dogs, Disease control

## Abstract

**Background:**

The prevalence of *Leishmania infantum* infection in clinically healthy dogs can be several times higher than that of clinical disease in endemic areas. Although treatment is not recommended in dogs with subclinical infection, these animals should be managed to prevent disease progression and parasite transmission to human beings or to other dogs. Dietary nucleotides and active hexose correlated compound (AHCC) have been shown to modulate the immune response. A recent study in dogs with clinical leishmaniosis receiving an initial 28-day course of methylglucamine antimoniate showed that six-month administration of a dietary supplement containing nucleotides plus AHCC achieves similar efficacy to allopurinol. Since the type of immune response plays a key role in the evolution of patients with leishmaniosis, the present study was aimed at evaluating the preventive effect of this supplement in avoiding or delaying disease progression in clinically healthy *Leishmania*-infected dogs.

**Methods:**

Forty-six dogs were included in this multicenter, randomized, double-blind, placebo-controlled trial. Dogs received once-daily oral administration of a placebo or a dietary supplement containing nucleotides plus AHCC. Disease progression was monitored throughout the study in both groups. At 0, 60, 180 and 365 days of treatment, clinical signs were evaluated using a validated clinical scoring system, and several analytes were measured from blood, urine, and bone marrow samples.

**Results:**

During the study, a significantly lower (*P* = 0.047) proportion of dogs changed their clinical status and became sick in the supplement group (3/20; 15%), compared to the placebo group (10/22; 45.5%). ELISA-determined antibody titers were significantly reduced compared to baseline at all time points with the supplement (*P* < 0.01), but not with the placebo. The mean clinical score of disease severity was significantly lower in the supplement group after 180 days (*P* = 0.014). No significant differences were observed for the other parameters. The dietary supplement was well tolerated.

**Conclusions:**

Oral administration of nucleotides plus AHCC for 365 days in clinically healthy *L. infantum*-infected dogs is safe, allows a significant reduction in anti-*Leishmania* antibodies, and leads to a lower disease progression rate, hence exerting a preventive effect.

## Background

*Leishmania infantum* is a protozoan parasite transmitted by the bite of a phlebotomine sand fly vector causing severe diseases in different mammalian hosts, including zoonotic leishmaniosis in humans and canine leishmaniosis (CanL) in dogs [1–3]. Subclinical infection, defined as a situation in which *Leishmania* infection is confirmed but clinical signs and/or clinicopathological abnormalities are not present, is more frequent than clinical disease [4–7]. Prevalence of infection can be as high as 50–80% in Mediterranean countries, while prevalence of disease varies from 2 to 5% [8–10]. The type of immune response raised against *L. infantum* determines whether a dog will develop clinical disease or remain in subclinical stage, and it also strongly affects the prognosis [1, 2, 11–14]. Clinically healthy *Leishmania*-infected dogs nowadays constitute a veterinary and public health concern because, although some of them may never develop clinical disease, they harbor *Leishmania* infection and thus represent a risk of parasite transmission [1, 14–17]. In dogs with subclinical infection, the ability to transmit the parasites to the vector has been proven using xenodiagnosis, although infectiousness appears to be higher in dogs with clinical leishmaniosis [18–22]. A higher parasite load in blood and skin has also been correlated with an increased infectiousness to the sand fly vector [23].

Current guidelines do not recommend treating dogs with subclinical infection because of the potential for promoting parasite resistance, and their management is currently based only on monitoring their clinical status and regular testing every three to six months to confirm seropositivity [1, 5, 12, 24]. It is, however, unlikely that treating only sick dogs will eventually reduce the prevalence of human or canine leishmaniosis as long as clinically healthy infected dogs maintain the infection in endemic areas [15]. Managing dogs with subclinical leishmaniosis is therefore an unresolved issue and innovative approaches are required.

CanL is endemic in the Mediterranean basin and South America, but in recent years, due to climate change, population instability, and globalization, a clear geographical expansion has become evident [6, 25–28]. Applying effective preventive measures is critical in order to reduce the dissemination of this important zoonosis. Effective control of CanL should address the vertebrate host, the vector and the parasite. Since dogs are the main natural reservoir host of infection for humans, this species should be the main target of control measures. Improving control of the spread of leishmaniosis in dogs may also result in a reduction in the number of cases in humans [2, 3, 12, 25, 29]. Newer areas in parasite control include the use of leishmanicidal or leishmaniostatic drugs in order to reduce parasite load in sick dogs, immunoprophylaxis through vaccination against *L. infantum*, and immunotherapy aimed at restoring an immune response so that the immune system becomes capable of controlling the infection. Until now, none of the available chemotherapies has reliably eliminated *Leishmania* infection and resistance to some of them have been reported in dogs [15, 30, 31]. Additionally, several commercial vaccine products have been licensed and marketed in Europe and Brazil, but their use is not yet widespread. Furthermore, current vaccines do not prevent establishment of infection, only disease progression and severity, and it is still necessary to further prove their long-term efficacy under field conditions [15, 31, 32]. Moreover, current diagnostic methods do not allow distinguishing between vaccinated dogs and naturally-infected dogs [2, 5, 13, 15, 33–36] except in dogs vaccinated with Letifend® (Laboratorios Leti, Barcelona, Spain) [37].

Immunotherapy is an area in which significant advances are being made in the control of *L. infantum* infection in dogs [15]. Domperidone, a dopamine D2 receptor antagonist, has been reported to reduce seroconversion rates in healthy seronegative infected dogs by enhancing their innate cell-mediated immune response [38]. In addition, intramuscular injection of a phospholinoleate-palmitoleate anhydride (P-MAPA) derived from *Aspergillus oryzae* in dogs with clinical leishmaniosis resulted in improvements of clinical signs and reduced parasite load in the skin [39]. Although these products appear to be safe, their prophylactic efficacy remains controversial [15]. Novel preventive treatment alternatives following this approach based on a modulation of the immune response are thus needed.

Nucleotides are low molecular weight biological molecules key to biochemical processes. Although under normal conditions de novo endogenous synthesis serves as the main nucleotide source, exogenous sources are essential to immune competence, intestinal development and recovery. Moreover, dietary intake becomes conditionally essential in certain situations where there is physiological stress and an increased demand for nucleic acid synthesis, including periods of immunosuppression, infection and certain disease states [40]. Dietary nucleotides have been shown to modulate the immune response, positively influencing lipid metabolism, immunity, and tissue growth, development and repair [41]. Active hexose correlated compound (AHCC) is a cultured extract of the mycelia of shiitake mushrooms (*Lentinula edodes*) used in humans for its ability to stimulate the immune system, especially enhancing cell immunity. This compound contains polysaccharides, amino acids, lipids, and minerals, and it is especially rich in α-glucans. One of its proposed potential mechanisms of action involves a toll-like receptor (TLR)-agonist activity of certain bioactive compounds found in AHCC [42–45].

A recent randomized controlled trial in dogs with clinical leishmaniosis receiving an initial 28-day course of methylglucamine antimoniate (MGA) showed that six-month oral treatment with nucleotides plus AHCC leads to efficacy similar to allopurinol, without promoting xanthinuria or urolithiasis. Thus, this combination could be a good alternative to the standard treatment, alone or together with conventional treatments, especially for CanL patients suffering allopurinol-related adverse events [46]. Based on these findings and on the nature of these compounds, we hypothesized that nucleotides plus AHCC might also serve as an appropriate treatment for preventing disease progression. The main objective of the present study was to evaluate the long-term effects of a dietary supplement containing nucleotides plus AHCC in clinically healthy infected dogs, and to assess whether the supplement might have a preventive effect protecting them from becoming sick.

## Methods

This was a multicenter, randomized, double-blind, placebo-controlled trial conducted in Spain. Client-owned dogs of any age, breed, or gender were recruited from 11 veterinary practices located in regions of Spain where leishmaniosis is endemic.

The main inclusion criteria were a positive serology result for *Leishmania* by enzyme-linked immunosorbent assay (ELISA) plus a positive cytology and/or PCR result obtained from bone marrow or lymph node aspirates, while not presenting with clinical signs or clinicopathological abnormalities associated with CanL. Samples were obtained at the initial screening visit. Dogs were excluded if they had been vaccinated against CanL, if they had received treatment with allopurinol, MGA, miltefosine, domperidone, ciclosporin, or glucocorticoids two months prior to the study outset, or if they were receiving any kind of special diet or supplements to improve their immune response [46–48]. Pregnant and lactating females were excluded. During the study, dogs were excluded if they showed clinical signs associated with CanL. Dogs could be withdrawn from the study at any time if they showed intolerance to the study compounds or severe clinical signs of the disease, or if requested by the owner.

Selected dogs were randomized using a computer-generated schedule into one of two treatment arms. Dogs in the supplement group were given a dietary supplement (Impromune®, Bioiberica S.A.U., Barcelona, Spain) in tablets containing 585 mg of a dietary nucleotides mix designed to mimic the nucleotide profile found in canine milk (Nucleoforce® Dogs, Bioiberica S.A.U.) plus 315 mg AHCC (Amino Up Chemical Co. Ltd., Sapporo, Japan) orally once daily for 365 days, following the dosage recommendations which provide a daily amount of 32 mg/kg nucleotides and 17 mg/kg AHCC. Dogs in the placebo group were administered inert microcrystalline cellulose tablets orally once daily for 365 days. Placebo tablets shared the same physical appearance as the supplement tablets. Treatment was started immediately after enrollment. During the study, all dogs were fed a regular diet, although different trademarks and formulations were allowed.

Clinical follow-up evaluations were conducted by each corresponding practitioner on days 0 (day of enrollment), 60, 180 and 365 of treatment. Each follow-up session consisted of a general physical exam and scoring for clinical signs associated with CanL using a scoring system which objectively quantifies the severity of disease from 0 to 55 [46]. Additionally, after 90, 135 and 270 days of treatment, owners were contacted by phone so that they could report any clinical signs that might require an additional visit to the veterinary practice.

The proportion of dogs in each group showing disease progression during the study was considered as the primary outcome. Disease progression was defined by the presence of clinical signs and/or clinicopathological abnormalities compatible with CanL, shifting from being clinically healthy into becoming a sick patient. This change in clinical status was used to attribute a possible preventive effect to the supplement. Dogs that became sick and showed disease progression during the study were excluded and only their data until the last visit before exhibiting disease progression were used for the final data analyses.

Blood samples were collected at 0, 60, 180 and 365 days of the trial to measure complete blood count (CBC), serum biochemistry, serum protein electrophoresis, and levels of antibody titers against *L. infantum* (Leiscan® *Leishmania* ELISA Test; Ecuphar, Spain; cut-off value of 1.1), CD4+ and CD8+ lymphocyte counts and several cytokines, namely interleukins 10 and 6 (IL-6 and IL-10), tumor necrosis factor alpha (TNF-α), and interferon gamma (IFN-γ). The CD4+/CD8+ ratio was calculated from CD4+ and CD8+ lymphocyte counts. Urinalysis, including the urinary protein/creatinine ratio (UPC), urinary density, and urinary sediment analysis (leucocytes, cylinders, bacteria, xanthine and struvite detection) was also performed initially, and at 60, 180 and 365 days after treatment onset on urine samples obtained by cystocentesis. Animals from each group and at each follow-up visit were classified according to International Renal Interest Society (IRIS) staging of chronic kidney disease based on blood serum creatinine levels and UPC values.

Bone marrow or lymph node aspirates were taken before (day 0) and after (day 365) treatment to evaluate parasite load by means of smear examination, nested PCR and real time-PCR (RT-PCR). Molecular diagnosis was performed on samples stored in 200 μl of buffer NET 10 (NaCl 10 mM, EDTA 10 mM, Tris 10 mM). The QIAamp® DNA Micro Kit (50) (Qiagen, Hilden, Germany) was used to obtain DNA according to the manufacturer’s instructions. *Leishmania* DNA was detected with PCR targeting internal transcribed spacers 1 and 2 as described by Kuhls et al. [49]. The PCR amplification product size was 280–330 bp. The parasite DNA load was quantified by amplification of a 200-bp kinetoplast DNA fragment using RT-PCR, as previously described [50], in a Corbett Rotor Gene 6000 thermal cycler (Qiagen). Results were expressed as parasites per ng of DNA.

During the follow-up period, any adverse events that could be related to the compounds, such as gastrointestinal disturbances or urinary abnormalities, were recorded.

Statistical analysis was performed by a biostatistician (JT), who was blinded to treatment assignment, using the software package SPSS Statistics v.22 (SPSS Inc., Chicago, IL, USA). A descriptive analysis of the data was performed according to the nature of the variables for each follow-up visit and assigned treatment. Quantitative variables are reported as the mean ± standard deviation (SD), and categorical variables as frequencies and percentages. Baseline differences were analyzed with a Student’s t-test for quantitative variables and Fisher’s exact test for categorical variables. Treatment effects were compared by analysis of covariance (ANCOVA) using baseline values as co-variables for quantitative variables. Fisher’s exact test was used for categorical variables. For quantitative variables, changes over time within each group were analyzed by repeated-measure analysis of variance (rmANOVA) and *post-hoc* tests of least significant difference (LSD). For categorical variables, changes over time were analyzed with McNemar’s test. The level of statistical significance was set at 5%.

## Results

**Fig. 1:**
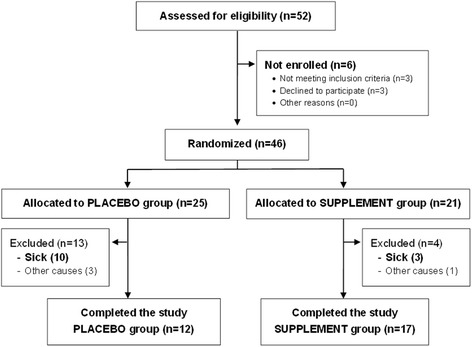
Flow diagram of the progress through the phases of the randomized clinical trial of the two study groups

A total of 52 dogs were assessed for eligibility. These dogs were recruited from a number of veterinary practices in Spain, namely HCV-Infectious Diseases Consultant UCM and CV Európolis, Madrid; CV Dinos, CV Dr. Bernal, CV Calas-Vet, CV Monteazahar, and CV Natura, Murcia; HV Althaía and CV San Francisco de Asís, Alicante; HV Canis, Girona; and CV San Jorge, Ibiza. Of the 52 dogs initially assessed, 46 met the inclusion criteria and were subsequently randomized into the two study groups (placebo group: *n* = 25; supplement group: *n* = 21). Of the 46 dogs included in the study, 26 (56.5%) [15/25 in the placebo group (60%) and 11/21 in the supplement group (52.4%)] had been treated with leishmanicidal and/or leishmaniostatic drugs at some point prior to eligibility assessment. Nevertheless, none of these dogs had received any leishmanicidal or leishmaniostatic drug within seven months prior to inclusion. Three dogs in the placebo group and one in the supplement group did not complete the study for reasons unrelated to disease progression and their data were not used for the results analysis. Two of these dogs from the placebo group died, one as a complication from a gastric dilation-volvulus syndrome and the other one due to sudden cardiorespiratory arrest with no apparent obvious cause at necropsy. None of these deaths were attributed to any reaction to the placebo tablets and no lesions compatible with clinical leishmaniosis were observed at necropsy. The other two cases, one in each group, did not complete the trial because the owners moved to different locations and were unable to carry on with the follow-up visits. A total of 12 dogs in the placebo group and 17 in the supplement group completed the clinical trial (Fig. 1). The baseline characteristics of the study population are provided in Table 1. Dog breeds included in the supplement/placebo groups were as follows: Boxer (2/3), German Shepherd (2/2), American Staffordshire (2/0), Rottweiler (1/1), Siberian Husky (1/0), Doberman (2/2), Brittany Spaniel (1/0), English Bulldog (1/0), Labrador Retriever (1/0), crossbreed (6/7), dogue de Bordeaux (1/0), Cocker Spaniel (1/1), Majorca Ratter (0/1), Bullmastiff (0/1), Pitbull (0/2), Bodeguero Andaluz (0/1), French Bulldog (0/1), English Setter (0/1), Saint Bernard (0/1), and Beagle (0/1). At baseline, there were no significant differences (*P* > 0.05) between study groups in mean age, age groups, breeds, gender distribution, temperature, or weight. The two groups were also initially matched in terms of clinical scores, and blood, bone marrow, and urine test results, and at study start there were no significant differences between groups for any of the studied parameters (*P* > 0.05).

**Table 1:** Baseline characteristics of dogs assigned to each study group, and homogeneity analysis data, expressed as mean ± standard deviation and frequencies (%)

Variable	Supplement group	Placebo group	*P*-value supplement *vs *placebo^a^
Gender: female, *n* (%)	10 (50)	7 (31.8)	0.346
Age: months	63.75 ± 33.78	67.36 ± 31.05	0.720
Age groups: *n* (%)			1.000
< 3 years	3 (15)	3 (13.6)	
3 to 8 years	13 (65)	15 (68.2)	
> 8 years	4 (20)	4 (18.2)	
Clinical score: points	0.80 ± 1.28	0.95 ± 1.73	0.756
ELISA serology: arbitrary units	3.31 ± 1.53	2.38 ± 1.46	0.054
RT-PCR: parasites/ng of DNA	0.22 ± 0.54	0.66 ± 1.89	0.420
Body temperature: °C	38.33 ± 0.49	38.40 ± 0.41	0.590
Weight: kg	27.90 ± 12.10	25.78 ± 15.11	0.621

^a^*P*-values were calculated with Student’s t-test for quantitative variables and Fisher’s exact test for categorical variables

**Fig. 2:**
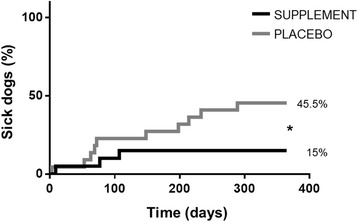
Changes in disease progression in patients from each group along the study. Data reported as percentage of clinically sick dogs in each study group. *P = 0.047, supplement *vs* placebo (Fisher’s exact test; OR = 4.722; CI = 1.068–20.887)

Changes regarding disease progression in each study group are shown in Fig. 2. During the study, 3 out of 20 dogs became sick and developed clinical leishmaniosis in the supplement group while 10 out of 22 in the placebo group did (15 *vs* 45.5%) (Fisher’s exact test; *P* = 0.047, OR = 4.722; CI = 1.068–20.887).

**Fig. 3:**
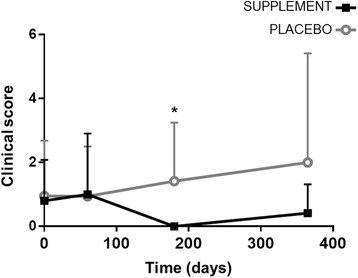
Changes produced in mean clinical score in dogs with CanL treated with supplement or placebo for 365 days. Data reported as mean ± SD. *P = 0.014, supplement *vs* placebo (ANCOVA; *F*_(1,23)_ = 7.068)

After 180 days of treatment a significantly lower clinical score was observed in the supplement group whilst adjusting for basal clinical score (mean ± SD 0.00 ± 0.00 *vs* 1.42 ± 1.83; ANCOVA; *F*_(1,23)_ = 7.068, *P* = 0.014). Over time, there were no significant changes (*P* > 0.05) in clinical score in any of the study groups (Fig. 3). No major variations between groups were seen in body weight or temperature over time in any of the study groups.

**Fig. 4:**
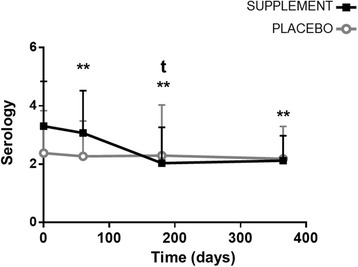
Changes produced in ELISA-determined antibody titers against *Leishmania* in dogs with CanL treated with supplement or placebo for 365 days. Data reported as mean ± SD. t, *P* = 0.056, supplement *vs* placebo (ANCOVA; *F*_(1,24)_ = 4.021); **P < 0.01 supplement *vs* supplement baseline (effect of time in rmANOVA; *F*_(2.154,23.692)_ = 9.639, *P* = 0.0007; LSD *post-hoc*)

Significant reductions in mean levels of ELISA titers were observed in the supplement group compared to baseline (3.31) (effect of time in rmANOVA; *F*_(2.154,23.692)_ = 9.639, *P* = 0.0007) after 60 (3.07; LSD *post-hoc*, *P* = 0.003), 180 (2.12; LSD *post-hoc*, *P* = 0.003), and 365 (2.04; LSD *post-hoc*, *P* = 0.002) days of treatment, while no significant changes were observed over time in the placebo group. When groups were compared, a trend towards significantly lower mean antibody titers (ANCOVA; *F*_(1,24)_ = 4.021, *P* = 0.056) was observed with the supplement after 180 days whilst adjusting for basal clinical score (Fig. 4).

No significant differences were observed between groups or over time in blood CD4+ and CD8+ levels, CD4+/CD8+ ratio, or cytokine levels (Table 2). No significant differences were seen between groups or over time in serum protein electrophoresis, CBC, biochemistry, or IRIS staging of chronic kidney disease (data not shown). Regarding parasite load, no significant differences (*P* > 0.05) were found over time or between groups in qualitative PCR, quantitative RT-PCR, or smear assessment. Likewise, there were no significant changes in urinary parameters within each group or between groups at any time point. Both the supplement and the placebo were well tolerated and no side effects related to these compounds were reported in any patient.

**Table 2:** Changes in lymphocyte counts and cytokine levels measured in each group during the study, expressed as mean ± standard deviation (ANCOVA)

Parameter	Supplement group	Placebo group	*P*-value supplement *vs* placebo (ANCOVA)
CD4+: %			
0 days	17.69 ± 15.37	13.94 ± 11.62	–
60 days	19.45 ± 15.14	14.69 ± 12.40	0.752
180 days	23.57 ± 12.45	19.89 ± 11.12	0.861
365 days	26.33 ± 9.95	24.07 ± 9.53	0.643
CD8+: %			
0 days	12.60 ± 13.48	7.65 ± 8.63	–
60 days	11.65 ± 10.40	8.16 ± 9.04	0.981
180 days	13.72 ± 12.39	7.76 ± 5.38	0.578
365 days	12.44 ± 6.76	10.76 ± 6.33	0.546
CD4+/CD8+ ratio			
0 days	2.01 ± 1.39	2.80 ± 1.90	–
60 days	2.41 ± 2.12	2.31 ± 1.33	0.394
180 days	3.10 ± 3.15	2.92 ± 1.50	0.238
365 days	3.21 ± 3.43	2.75 ± 1.11	0.721
IL-6: pg/ml			
0 days	382.91 ± 940.53	269.95 ± 1054.1	–
60 days	138.65 ± 371.02	393.12 ± 1424.6	0.165
180 days	192.38 ± 456.17	118.67 ± 184.77	0.728
365 days	314.92 ± 1024.5	34.63 ± 55.48	0.612
IL-10: pg/ml			
0 days	22.69 ± 76.01	17.92 ± 63.47	–
60 days	7.52 ± 12.67	38.51 ± 147.25	0.208
180 days	4.33 ± 4.34	69.35 ± 231.80	0.172
365 days	6.53 ± 10.01	2.68 ± 0.74	0.282
TNF-α: pg/ml			
0 days	123.14 ± 292.94	81.58 ± 308.77	–
60 days	40.47 ± 95.60	112.17 ± 397.27	0.150
180 days	59.21 ± 144.19	44.82 ± 72.46	0.811
365 days	73.11 ± 233.46	13.23 ± 19.81	0.597
IFN-γ: pg/ml			
0 days	15.97 ± 35.87	3.49 ± 8.12	–
60 days	7.04 ± 13.64	1.39 ± 2.33	0.264
180 days	0.81 ± 0.84	0.84 ± 0.42	0.980
365 days	4.58 ± 13.43	1.30 ± 0.97	0.544

## Discussion

Dogs are the main reservoir of *Leishmania* infection in humans; hence, the proposed unified medical-veterinary strategy of controlling the infection in the canine population in order to reduce the availability of parasites to sandflies and subsequently decrease the incidence of human and canine leishmaniosis [3, 6, 15, 29, 38, 51]. Defining appropriate management of healthy infected dogs is of essential importance because, although treating them is not recommended, they harbor infection and represent a risk of parasite transmission in endemic areas as well as a threat for further spreading in non-endemic regions [1, 24]. In an attempt to provide new tools to better approach this therapeutic dilemma, we evaluated the effects of a dietary supplement containing nucleotides and AHCC in dogs with subclinical leishmaniosis.

Clinically, all dogs enrolled in this study were initially in subclinical stage. During the study, a significantly lower rate of disease progression was observed in the supplement group, which might indicate a possible preventive effect in clinically healthy infected dogs. The use of a systematic and comprehensive clinical score [46] allowed the quantification of changes in the main clinical signs associated with CanL [11, 52] in each group as well as the subsequent detection of a significantly lower clinical score in the supplement group after six months. This is in accordance with a previous report in sick dogs with leishmaniosis that improved clinically after receiving the same dietary supplement also during six months [46].

The humoral immune response in this study was examined through measurement of antibody titers with ELISA serology testing. The oral administration of the supplement led to a significant reduction in antibody titers at all time points, unlike what happened with the placebo. In effect, the combined use of this supplement with MGA had already shown such benefit on decreasing antibody titration after a six-month treatment period [46]. The present study adds further support to this effect in dogs receiving nucleotides plus AHCC for a whole year as a sole therapy.

The lack of significant changes over time or between groups in parasite load, cytokine levels or lymphocyte counts might be explained by the fact that disease severity of dogs included in the study was considered low [14], and that data from dogs which had exhibited a clinical worsening from the time point in which they showed disease progression was not used for the final data analyses.

Abnormalities in urinary parameters were not observed in this study in any of the groups. Dogs with leishmaniosis managed following the “first line treatment” [52] based on the combined use of antimonials and allopurinol (considered the most effective treatment protocol against CanL [35]) might eventually develop xanthinuria as a side effect of allopurinol administration, which can lead to renal mineralization and even urolithiasis [53–57]. In a recent study, the administration of nucleotides and AHCC for six months in sick CanL patients did not enhance the development of xanthinuria [46]. The present study now allows us to also state that administering this supplement does not promote any type of crystalluria after a one-year administration period in healthy CanL patients, thus further supporting the safety of the combination.

Also regarding safety, this is the first report to evaluate the effects of the oral administration of nucleotides plus AHCC in dogs for one year, confirming the safety of this combination when used long-term. The use of other immunomodulatory compounds, such as domperidone and P-MAPA, has been proposed in the past given the contribution of cellular immunity to leishmaniosis progression [15, 38, 39, 58]. Domperidone administration has been associated with some side effects, but they are not usually severe (mild galatorrhea or mild gastrointestinal disturbances) [38]. On the other hand, after the oral administration of nucleotides plus AHCC in this study or in a previous study [46] there were no associated side effects and it can be considered safe. P-MAPA and AHCC are both processed natural extracts of fungal origin [39, 43]. Bioactive compounds found in mushroom extracts have been shown to possess immunomodulating properties [59], which could explain in part the reported benefits of administering these compounds to CanL patients [39, 46].

A TLR-mediated activity might explain the effects observed in CanL patients receiving a combination of nucleotides and AHCC orally. In canine *L. infantum* infection, TLRs play a key role as part of the innate immune response against the parasite. More specifically, TLR2 is upregulated in sick dogs, and TLR2 transcription decreases as patients respond positively to therapy [60]. It has been proposed that both nucleotides [40] and AHCC might interact with TLR signaling. AHCC may prime the TLR-2 and TLR-4 gate at the intestinal epithelium, probably through recognition of non-pathogenic food-associated molecular patterns (FAMPs) [44]. This mechanism has been attributed to bioactive compounds found in certain mushroom extracts and yeast-derived compounds, such as nucleotides. However, this was not specifically investigated in the present work, therefore further studies are needed in order to confirm this hypothesis and to better characterize the mechanisms of action that drive the modulation of the immune response exerted by this supplement which results in clinical benefits in CanL patients.

This study provides a novel therapeutic alternative for the management of clinically healthy dogs with leishmaniosis based on immunomodulation of the disease through nutrition. As reported herein, one-year oral administration of nucleotides plus AHCC positively influenced disease progression, suggesting the use of this supplement for preventive purposes. Nevertheless, the present study has some limitations. First, the sample size was small, which might have affected the significance of some of the results. Secondly, we cannot rule out the effect of diet on the immune system of the dogs in the study because, although they all received a regular diet, different trademarks and formulations were used. Lastly, the washout periods for leishmanicidal and leishmaniostatic drugs defined as exclusion criteria might have been too short, therefore future studies should use longer periods. However, although we cannot completely rule it out, a possible interference of a prior treatment on the observations made during the study is not very likely given that none of the included dogs had received any leishmanicidal or leishmaniostatic drug within seven months prior to inclusion.

Our results, together with the above-mentioned limitations, open the door for further investigations. Although it appears that the supplement acts on both the humoral (seen as serological improvements) and cellular immune responses [46], the mechanism of action is not yet fully known. Studies evaluating the specific actions in this sense of nucleotides and AHCC alone or in combination are needed. Additionally, interventions aimed at modulating the immune response, such as the one described herein, could be used in future studies assessing possible enhancing effects of such compounds on the efficacy of vaccines against *Leishmania*. Additional clinical trials with a larger sample sizes, a proper clinical staging of patients [1], longer follow-up, and also combining the supplement with other leishmanicidal drugs would be desirable in order to further confirm our observations.

Based on findings from this study and also on prior observations in sick CanL patients [46], a combination of nucleotides and AHCC might become a useful tool either as sole therapy in clinically healthy infected dogs or as an adjunctive treatment to already existing therapies in sick dogs in order to obtain better clinical efficacy and perhaps be able to reduce dosages of anti-*Leishmania* drugs and thereby lower the risk of developing resistances. Finally, our results lend support to the inclusion of such supplement among the already existing recommendations for the prevention of leishmaniosis [15] and to the consideration of this supplement as part of the multi-modal treatment approach described in the current guidelines for the management of CanL [1, 24]. Eventually, a combination of nucleotides and AHCC might contribute to finding a solution for the currently unresolved issue of being unable to treat clinically healthy infected dogs.

## Conclusions

The oral administration of a dietary supplement containing nucleotides and AHCC for 365 days in clinically healthy *L. infantum*-infected dogs is safe, and it leads to significant reductions in ELISA serology titers of antibodies against *Leishmania* and the rate of disease progression. These findings confirm the preventive effect of this immunomodulatory dietary supplement in CanL clinically healthy dogs, reducing their progression into sick patients when compared to non-supplemented dogs.

## Abbreviations

AHCC: Active hexose correlated compound
ANCOVA: Analysis of covariance
CanL: Canine leishmaniosis
CBC: Complete blood count
ELISA: Enzyme-linked immunosorbent assay
FAMPs: Food-associated molecular patterns
IFN-γ: Interferon gamma
IL-10: Interleukin 10
IL-6: Interleukin 6
IRIS: International Renal Interest Society
LSD: Least significant difference
MGA: N-methylglucamine antimoniate
P-MAPA: Phospholinoleate-palmitoleate anhydride
rmANOVA: Repeated-measures analysis of variance
RT-PCR: Real time-PCR
TLR: Toll-like receptor
TNF-α: Tumor necrosis factor alpha
UPC: Urinary protein/creatinine ratio
